# Oxidative Stress Is Increased in Combined Oral Contraceptives Users and Is Positively Associated with High-Sensitivity C-Reactive Protein

**DOI:** 10.3390/molecules26041070

**Published:** 2021-02-18

**Authors:** Sabina Cauci, Serena Xodo, Cinzia Buligan, Chiara Colaninno, Mattia Barbina, Giuseppe Barbina, Maria Pia Francescato

**Affiliations:** 1 Department of Medicine, School of Medicine, University of Udine, 33100 Udine, Italy; chiaracolaninno1@gmail.com (C.C.); mattiabarbina@gmail.com (M.B.); mariapia.francescato@uniud.it (M.P.F.); 2 Gynecology and Obstetrics Clinics, Maternal and Child Department, Santa Maria della Misericordia University-Hospital, 33100 Udine, Italy; serenaxodo@yahoo.it; 3 Dermatology Unit, Santa Maria della Misericordia University-Hospital, 33100 Udine, Italy; C.Buligan@gmail.com; 4 Clinical Analysis Laboratory, Department of Laboratory Medicine, Institute of Clinical Pathology, Santa Maria della Misericordia University-Hospital, 33100 Udine, Italy; barbinagiuseppe@gmail.com

**Keywords:** lipid peroxidation, free radicals, inflammation, contraceptive pill, thromboembolism, hormonal contraception, estrogen, progestin, third generation pill, alimentary habits

## Abstract

Information concerning the mechanisms underlying oxidative stress and low-grade inflammation in young healthy women predisposing eventually to future diseases is scarce. We investigated the relationship of oxidative stress and high-sensitivity C-reactive protein (hsCRP) in fertile-age women by oral combined contraceptive (OC) use. Caucasian Italian healthy non-obese women (*n* = 290; 100 OC-users; 190 non-OC-users; mean age 23.2 ± 4.7 years) were analyzed. Blood hydroperoxides, as oxidative stress biomarkers, were assessed by Free Oxygen Radical Test (FORT). Serum hsCRP was determined by an ultra-sensitive method (hsCRP). Markedly elevated oxidative stress (≥400 FORT Units) was found in 77.0% of OC-users and 1.6% of non-OC-users, odds ratio (OR) = 209, 95% CI = 60.9–715.4, *p* < 0.001. Elevated hsCRP levels ≥ 2.0 mg/L, considered risky for cardiovascular diseases (CVDs), were found in 41.0% of OC-users and 9.5% of non-OC-users, OR = 6.6, 95%CI 3.5–12.4, *p* < 0.001. Hydroperoxides were strongly positively correlated to hsCRP in all women (r_s_ = 0.622, *p* < 0.001), in OC-users (r_s_ = 0.442, *p* < 0.001), and in non-OC-users (r_s_ = 0.426, *p* < 0.001). Women with hydroperoxides ≥ 400 FORT Units were eight times as likely to have hsCRP ≥ 2 mg/L. In non-OC-users only, hydroperoxides values were positively correlated with weight and body mass index, but negatively correlated with red meat, fish and chocolate consumption. Our research is the first finding a strong positive correlation of serum hydroperoxides with hsCRP, a marker of low-grade chronic inflammation, in young healthy women. Further research is needed to elucidate the potential role of these two biomarkers in OC-use associated side-effects, like thromboembolism and other CVDs.

## 1. Introduction

The biological pathways and the biomarkers associated with the side effects of hormonal contraception remain elusive and require additional research. Large studies and meta-analysis have assessed the association of oral combined contraceptive (OC) use with major complications, mainly cardiovascular diseases (CVDs) like arterial and venous thromboembolism (VTE), myocardial infarction, ischemic stroke, pulmonary embolism, peripheral arterial disease, and sudden cardiac death [1,2,3,4,5,6,7,8,9,10,11] and also some types of cancer including breast, uterine cervix, as well as liver and bile duct cancers [12,13,14]. Moreover, a higher risk for preterm birth in women who had used OCs before pregnancy compared with a control group was demonstrated [15]. Some evidence shows also a correlation of OC use with mood disorders and depression [16,17].

Presently, limited research assessed oxidative stress [18,19,20,21,22,23] and low-grade inflammatory status [24,25,26,27,28,29,30,31] in pre-menopausal women OC-users, two conditions that have been independently implicated in several pathologies, including CVDs and cancer. Rather surprisingly no study examined the relationship between oxidative stress and low-grade inflammatory status in a sample of childbearing age women according to OC use. Importantly, both oxidative stress and low-grade inflammatory status can, at least in part, be modulated by lifestyles [25,29,32,33,34,35,36,37].

The overproduction of reactive oxygen species (ROS), like superoxide anion (O_2_^•−^), hydroxyl radical (HO^•^), peroxide (O_2_^2−^), when not balanced by the antioxidant defence (including enzymatic and non-enzymatic molecules) causes oxidative stress [38]. Free radicals can attack various molecules (namely lipids, nucleic acids, proteins, etc.) becoming deleterious for the human body because of the formation of altered molecules [38]. It is well established that oxidative stress has major roles in the pathogenesis of several diseases such as inflammatory, cardiovascular, muscular, and neurodegenerative diseases and cancer [38]. However, ROS have also physiological positive roles as free radicals contribute in signalling devoted to regulate cell functions and inflammatory responses to microorganisms and xenobiotics [38].

Various biomarkers have been used to evaluate oxidative stress in fertile age women of the general population [19,20,21,22,23,33,35] suggesting an increase associated to hormonal contraception. In young female athletes a recent research showed increased oxidative stress in OC-users compared to non-OC-users [18].

On the other hand, there is some evidence that OC use raises the chronic low-grade inflammatory status as assessed by the increase of high-sensitivity C-reactive protein (hsCRP) [24,25,26,27,28,31]. Elevation of hsCRP in young women [16] implies several potential adverse effects including CVD risk [39,40], endothelial damage, myocardial infarction, and thromboembolic events [2]. In women, for assessment of CVD risk stratification, the hsCRP cutoff concentrations of 2 mg/L and 3 mg/L were frequently used as risky, and <0.5 mg/L as no risky/protective [41].

The relation of C-reactive protein to oxidative stress was previously investigated in some pathologic conditions like hypertension [42], renal failure [43] or dilated cardiomyopathy [44]. However, this relationship was never investigated in healthy women. Progress in the understanding of the regulatory mechanisms underlying oxidative stress and chronic low-grade inflammation can furnish insights for the prevention of OC side effects in women. Moreover, it is interesting to establish whether the two serum biomarkers levels are potentially modifiable by lifestyle and alimentary habits of young women.

The general aim of the study was to investigate whether serum hydroperoxides (as indicator of oxidative stress) and hsCRP (as marker of chronic low-grade inflammation) levels were interrelated in young women also according to OC use.

## 2. Materials and Methods

### 2.1. Design and Setting of the Study

The study was conducted on healthy Italian adult women enrolled in the years up to September 2019; none of the volunteers were athletes, to avoid confounding effects of physical exercise [25].

All participants attended once the Medical Department laboratory (University of Udine, Italy) early in the morning (8–10 am) after 12 h fasting. A finger capillary and a venous blood withdrawal [24,25] was thus taken on seated subjects [18,45]. The day before, volunteers were asked to refrain from alcohol and supplement consumption as well as from physical activity. Menstrual cycle phase was not accounted for; however, menstruation bleeding days were avoided (days 1–7 of menstrual cycle) because during those days oral contraception consists of drug discontinuation or placebo use. Clinical, demographic, and habit data were blinded to personnel executing the blood collection and samples measurements.

### 2.2. Population

Women (age range 18–46 years) were recruited consecutively; announcements at Udine University were used to enroll the volunteers. All study participants filled questionnaires and volunteered for a venous and a finger capillary blood withdrawal. The inclusion criteria were: (a) no acute (current/recent) infection or chronic disease such as diabetes mellitus, autoimmune diseases, thyroid or cardiovascular disease, or any tumor [24,25]; (b) absence of pain from whatever cause [46]; (c) no assumption of anti-inflammatory drugs or antibiotics in the last 2 weeks [24,25], (d) no hormonal drugs other than monophasic combined contraceptive pills [24,25], (e) not lactating, pregnant, or postmenopausal [47]; (f) no present or past menstrual dysfunction [48]; and (h) no major sleeping disorders [18]. Eligible women for inclusion in the OC-users group were those using a monophasic combined oral contraceptive for at least 3 months, whereas for inclusion in the non-OC-users control group, women had never used hormonal contraception or had discontinued since at least 3 months any hormonal treatment [18,25]. Three-hundred women were examined for eligibility; at the time of blood withdrawal, 10 women were excluded due to acute disease potentially affecting the inflammatory status. A total of 290 participants were thus included in the study (*n* = 100 OC-users and *n* = 190 non-OC-users).

Demographic factors, medical history and lifestyle habits (including smoking) were collected by a questionnaire. Moreover, a 2 weeks long diary allowed to collect alimentary habits in the form of daily number of servings as already described [18]. Alimentary supplements consisted of a wide variety of commercial products containing variable amounts of single or mixed compounds, assumed by women occasionally, intermittently, or continually. Thus, alimentary supplements were not categorized into specific subgroups, and a categorical variable (yes or no) designed “supplement use” was employed for statistical analysis. Body mass index (BMI) was calculated as body mass (kg) divided by the square of stature (m).

### 2.3. Ethical Considerations

Written informed consent was obtained from each participant before enrolment and no compensation or incentive was paid to the participants for this study. The study was approved by the Institutional Ethical Committee of the Department of Medicine (University of Udine, number of ethical approval 554), and was conducted according to the rules of the Declaration of Helsinki (1975).

### 2.4. Oxidative Stress Assessment

Blood hydroperoxides, mainly consisting of lipid hydroperoxides [18,45], were determined in 20 µL of capillary blood using the Free Oxygen Radical Test (FORT assay; Callegari, Parma, Italy), a 6 min long colorimetric assay based on the ability of transition metals to catalyze the breakdown of hydroperoxides (ROOH) into radicals, according to the Fenton reaction [45]. Results were expressed as FORT Units, whereby 1 FORT Unit corresponded to 0.26 mg/L H_2_O_2_ [45]. Variations of the intra- and inter-assay were both <5.0%. The range limits of measurable values were ≤160 and ≥600 FORT Units. Four non-OC-users had FORT values ≤ 160 and were set at 160 FORT Units, whereby 9 OC-users had ≥600 FORT Units and were set at 600 FORT Units for statistical calculations.

The threshold of 310 FORT Units was used as high cut-off level of hydroperoxides [18], and a value of 400 FORT Units was employed as very high threshold level of oxidative stress [49].

### 2.5. C-Reactive Protein Measurement

Just after collection, venous blood was processed for serum separation as already described [25].

Serum hsCRP was measured on Olympus AU5400 biochemistry analyzer (Olympus Diagnostic Systems Group, Brea, CA, USA) using an ultra-sensitive immunoturbidimetric assay (Olympus System CRP latex). Detection limit of the assay was ≤0.1 mg/L. Samples with values below the detection limit were set at 0.1 mg/L for statistical calculations. The intra- and inter-assay variations were 1.1%, and 4.0%, respectively. Stratification of hsCRP levels were performed at 0.5 mg/L as no risky level, and 2.0 mg/L and 3 mg/L as risky levels for future CVD, as described [24,39,41].

### 2.6. Statistical Analysis

Normality of distribution of variables was assessed by the Kolmogorov-Smirnov test. Gaussian distributed data were shown as mean ± standard deviation (SD). The FORT and hsCRP data were not normally distributed, thus, their values were shown as median and interquartile range (IQR, 25th to 75th percentile) and non-parametric tests were used. Continuous variables were compared by the Mann-Whitney U-test. The comparison of proportions between groups was assessed by Pearson’s X^2^-test that was appropriate for all the categorical variables except for “nulliparity” for which Fisher’s exact test was used. Crude odds ratios (ORs) and 95% confidence intervals (CIs) were evaluated for categorical variables. Logistic regression was performed to evaluate the difference in oxidative stress between groups after adjustment for: age, BMI, smoking, supplement use, chocolate, fresh vegetable, fruits, red meat and fish servings per week. Bivariate relationships were evaluated by Spearman Rho test (r_s_). All tests were 2-sided. Statistical significance was set at *p* values < 0.05. At an alpha level of 0.01, we had over 95% power to detect a difference in the proportion cases with hsCRP ≥ 2 mg/L between the OC-users and the non-OC-users groups and to find a correlation between hsCRP and FORT Units with r_s_ > 0.4. Statistical analysis was performed using the Statistical Package for Social Sciences (SPSS for Windows, SPSS Inc., Chicago, IL, USA).

## 3. Results

On average, the 290 study women were 23.2 ± 4.71 year-olds, had BMI 21.2 ± 2.31 kg/m^2^, 97.9% were nulliparous, mostly enrolled at or employed by Udine University, 82.8% had university level education, 16.6% were smokers, and all had a middle-class socioeconomic status. Main demographic and lifestyle characteristics and continuous values of oxidative stress and hsCRP of the 100 OC-users and the 190 non-OC-users are summarized in Table 1.

A normal weight range (BMI 18.0–25.0 kg/m^2^) was found in 91.4% of women, 9 women were underweight (3.1%, BMI < 18.0 kg/m^2^), and 16 were overweight (5.5%, BMI > 25.0 kg/m^2^); none of the women, however, had a BMI ≥ 30.0 kg/m^2^. OC-users had slightly more frequently a university education (89.0%) than non-OC-users (79.5%), *p* = 0.041 (Table 1). However, most of the studied parameters were not significantly different between OC-users and non-OC-users including smoking habits, coffee and nutritional supplement use. In our sample, women smoke on average less than 1 cigarette per day. Only one woman smoked 26 cigarettes per day, none of the other smokers smoked more than 15 cigarettes per day.

Table 1 shows median (25th to 75th percentile) values of hydroperoxides that were 1.7-fold higher in OC-users compared to non-users (median 472 vs. 270 FORT Units, *p* < 0.001). Values of hsCRP were 3.7-fold higher in OC-users compared to non-users (median 1.31 vs. 0.35 mg/L, *p* < 0.001).

### 3.1. Combined Oral Contraception

A hundred women were using OC from 3 to 144 months. Continuous values of FORT Units and hsCRP concentration were not correlated with months of OC use (*p* > 0.05 for both).

OCs had different formulations but all were monophasic combined pills. Pills had variable progestin components: gestodene (43.0%), drospirenone (24.0%), desogestrel (12.0%), levonorgestrel (10.0%), cyproterone (4.0%), dienogest (3.0%), clormadinone (2.0%), and nomegestrolo acetate (2.0%). Overall, among the 100 OC-users second generation pill preparations (containing levonorgestrel) were used by 10.0%, third generation pill preparations (having gestodene or desogestrel as progestin) by 55.0%; and fourth generations pills (containing drospirenone, cyproterone, dienogest, clormadinone and nomegestrolo acetate) by 35.0% of women. Preparations containing progestogens with the highest risk of VTE according to recent evidence [5] i.e., those including desogestrel, cyproterone, and drospirenone were used by 40.0% of OC-users.

Table 2 shows continuous values of hydroperoxides (FORT Units) and hsCRP (mg/L) in each generation pill users. Use of second generation pill was associated with lower values of hydroperoxides than third (*p* = 0.009) and fourth (*p* = 0.008) generation; conversely, third and fourth generation did not differ. Concentrations of hsCRP did not vary significantly among groups.

Moreover, by analysis of each generation pill vs. others, second generation pill users compared to all other pills showed lower hydroperoxides (median 366 (IQR 332–434) vs. 484 (IQR 421–542) FORT Units, *p* = 0.006), whereas did not differ for hsCRP values (*p* = 0.509). Comparison of 55 users of third generation pills with other pill preparations did not show significant differences for hydroperoxides (*p* = 0.997) and hsCRP (*p* = 0.923). Fourth generation pill users were not different from the other pills in hydroperoxides (*p* = 0.083) and hsCRP (*p* = 0.753). However, by comparing the 40 users of OC at high risk of thromboembolism (i.e., those containing desogestrel, cyproterone, and drospirenone) with all the other remaining pills, oxidative stress was higher (median 492 (IQR 442–576) vs. 444 (IQR 367–502) FORT Units, *p* = 0.003), although hsCRP did not differ (*p* = 0.198).

### 3.2. Stratified Values of Oxidative Stress and hsCRP

Table 3 shows the comparison of OC-users and non-OC-users according to categorized values of hydroperoxides and of hsCRP.

A marked difference was noted for hydroperoxides values ≥ 310 FORT Units, which were detected in 92.0% of OC-users compared to 23.7% of non-OC-users, crude OR = 37.1, *p* < 0.001, adjusted OR = 100, *p* < 0.001. Very high levels of hydroperoxides ≥ 400 FORT Units were observed in 77.0% of OC-users compared to 1.6% of non-OC-users, crude OR = 209, *p* < 0.001, adjusted OR = 342, *p* < 0.001.

Regarding stratified hsCRP levels, the percentage of subjects with risky hsCRP concentrations was significantly higher in OC-users compared to non-OC-users; for the cutoff value of ≥2 mg/L crude OR 6.64, adjusted OR 11.1, and for the cutoff value of ≥3 mg/L crude OR 6.71, adjusted OR 8.05. Conversely, non-OC-users were much more likely to show protective hsCRP < 0.5 mg/L values compared to OC-users (OR 8.60; *p* < 0.001). Very few women (*n* = 6) had hsCRP ≥ 10 mg/L, 5 among the OC-users, and 1 among the non-OC-users. No woman had hsCRP ≥ 20 mg/L.

Women having elevated hsCRP values were much more likely to show elevated oxidative stress (Table 4). Specifically, among a total of 59 women having risky hsCRP ≥ 2 mg/L, 49 (83%) had FORT ≥ 310 Units and 38 (64%) had FORT ≥ 400 Units; furthermore, among a total of 46 women having hsCRP ≥ 3 mg/L, 39 (85%) had FORT ≥ 310 Units and 32 (70%) had FORT ≥ 400 Units. On the contrary, women with low protective levels of hsCRP (<0.5 mg/L) were much less likely to have elevated oxidative stress. Indeed, among the 134 women having protective hsCRP < 0.5 mg/L, only 26 (19%) had FORT ≥ 310 Units and 7 (5.0%) had FORT ≥ 400 Units.

By considering the two elevated thresholds of oxidative stress, namely FORT ≥ 310 or ≥400 Units (Table 4), women were roughly 8-fold more likely to have elevated hsCRP levels.

In a refined analysis, stratified levels of FORT ≥ 400 Units and hsCRP concentrations were examined separately in 100 OC-users. Among 77 OC-users with FORT ≥ 400 Units 37 (48%) had hsCRP ≥ 2 mg/L and 31 (40%) had hsCRP ≥ 3 mg/L, whereas among 23 OC-users with FORT < 400 Units only 4 (17.4%) had hsCRP ≥ 2 mg/L and 2 (8.7%) had hsCRP ≥ 3 mg/L, with OR = 4.39, 95%CI 1.37–14.1, *p* = 0.009, and OR = 7.08, 95%CI 1.55–32.4, *p* = 0.005, respectively. OC-users with FORT ≥ 400 Units were less likely to have CRP < 0.5 mg/L, OR = 0.16, 95%CI 0.05–0.49, *p* = 0.002.

Table 5 summarizes the correlations of hydroperoxides continuous values (in FORT Units) with other biomarkers in all 290 study women, and separately in the 100 OC-users and 190 non-OC-users. FORT Units were highly positively correlated with hsCRP concentrations with *p* < 0.001 in each of the 3 groups (r_s_ = 0.622, 0.442, 0.426, respectively). A positive association of FORT Units with body weight was found in non-OC-users only (*p* = 0.005). Oxidative stress showed significant positive associations with BMI in all women (*p* = 0.047) and non-OC-users (*p* = 0.001). Concerning the alimentary habits, hydroperoxides showed significant inverse associations with red meat, fish and chocolate servings in all women (*p* = 0.004, 0.027, 0.041, respectively) and non-OC-users (*p* = 0.007, 0.045, 0.028, respectively). Finally, we found an inverse correlation of hydroperoxides in all women with fruits (*p* = 0.035) and total meat servings (*p* = 0.029). Remarkably, in OC-users none of the examined demographic, lifestyle/alimentary habits were associated with FORT Units, with exception of hsCRP.

## 4. Discussion

The main outcomes of the present study are: (1) Elevated oxidative stress levels were found in non-athlete OC-users belonging to the general population of healthy young women; (2) OC-use resulted in chronic inflammation, confirming previous studies performed in lower number of women or different ethnic groups; (3) Oxidative stress and hsCRP were strongly correlated; (4) Body mass (as well as BMI) and some alimentary habits modulated oxidative stress in non-OC-users only.

### 4.1. Oxidative Stress

Oxidative stress was evaluated by an assay measuring hydroperoxides (expression mainly of lipid peroxidation) [33,45,50], that constitutes only one of the possible indirect markers to assess oxidative stress status [51]; however, the FORT assay has been validated to assess oxidative stress in human blood by various recent studies [18,32,45,52]. Specifically, the FORT assay has been validated for clinical use in women and has the advantage to be easily used in outpatient facilities [32,52]. Lewis, et al. [52] determined a value for individual biological variations of the FORT assay equal to 5.0%.

We found a remarkable elevated frequency of high oxidative stress levels in healthy OC-users; by the cutoff value of ≥310 FORT Units 92.0% (crude OR = 37, adjusted OR = 100 compared to non-OC-users), and by the cutoff value of ≥400 FORT Units 77.0% (crude OR = 209, adjusted OR = 342). The median of continuous FORT Units was almost 2-fold higher in OC-users than in non-OC-users. In our study, among OC-users oxidative stress was not associated to their lifestyles and alimentary habits according to the study parameters. It appears that pro-oxidant effects of OCs likely overwhelmed antioxidant effects of lifestyle/alimentary good habits in OC-users. At variance, oxidative stress in non-OC-users was negatively associated with some alimentary habits, specifically chocolate, red meat and fish servings per week. Our findings are consistent with the known antioxidant properties of some foods [53,54,55]. It is to mention that in our study fruits consumption had a negative correlation with oxidative stress in all the 290 women, however, in the distinct subgroups of 100 OC-users and 190 non-OC-users such negative correlation did not reach statistical significance, thus, fruits effects will require further research [54].

We did not found oxidative stress association with smoking and supplement consumption in the study women. The number of cigarettes smoked, however, was very low (on average <1 cigarette/day), moreover participants were asked to avoid supplements use in the day before blood testing. Interestingly, a study [32] performed in post-menopausal women showed that folate administration, in the form of 5-methyltetrahydrofolate (5-MTHF), can reduce blood oxidative stress as assessed by FORT values reduction. Particular effects of supplements and/or drugs will require specific further research [56].

Elevated BMI is known to increase systemic oxidative stress in the general population [57], and in active adults [34]. Confirmatory with other studies, we found that BMI was positively correlated to the FORT levels in non-OC-user women, but not in OC-users. A similar finding was seen previously in female athletes [18]. Apparently, the strong OC induction of oxidative stress cancelled benefits deriving from reduced BMI.

Results of our current study performed on the general female population concur with a previous investigation evaluating oxidative stress in female athletes [18] founding that 42 OC-users had significantly higher hydroperoxides than 102 non-OC-users (OR = 42, 95%CI = 12–149, *p* < 0.001). However, that study did not measured hsCRP [18].

Consistently with the present study, a research conducted in 40–48 years old Belgian women found a significant increase of lipid peroxides in 209 OC-users compared to 119 non-users of contraception [23]. Additionally, a study comparing 32 OC-users with 30 non-OC-users found increased lipid peroxides (+176%, *p* < 0.001) and oxidized LDLs (+45%, *p* < 0.002) in the former group of women [21].

Interesting studies [22,33], investigating the time-course of hydroperoxide elevation in women users of a low estrogen dose pill containing drospirenone, demonstrated that oxidative stress increased significantly after only one week of OC use, remained constantly elevated during OC use, and returned to basal levels within one week of OC discontinuation, thus suggesting a causative role of OC use in increasing oxidative stress [33].

Mechanisms leading to elevation of hydroperoxides by OC are still not definitively characterized [33], however, some evidence point to oxidative hepatotoxicity of OC [12]. P450 cytochromes (CYPs) catabolizing exogenous hormones can cause increased ROS production [58] and, in turn, hyper-production of free radicals could provoke depletion of antioxidant defenses such as depletion of reduced glutathione [33,35]. However, the role of estrogens and progestogens in OC induced oxidative stress is still debated [21,33,59]. An in vitro study showed that beta-estradiol treatment of cells was cytotoxic through oxidative stress inducing a significant increase in lipid peroxidation [60].

By recent evidence tissue redox status is adequately reflected by redox blood biomarkers [61], thus, the increased oxidative stress measured in blood associated to OC use likely parallels increased free radicals also in several body organs [62].

### 4.2. C-Reactive Protein

In the present study, OC-use significantly increased all risky levels of hsCRP, while provoking a loss of the protective levels below 0.5 mg/L. Specifically, OC-users were more likely to have hsCRP levels ≥ 2 mg/L (crude OR = 6.64, adjusted OR = 11.1) and ≥3 mg/L (crude OR = 6.71, adjusted OR = 8.05) than non-OC-users, two cutoff values associated with CVD risk. These results are consistent with previous Italian studies performed in 77 third generation pill OC-users (OR = 4.04; 95% CI 1.99–8.18, *p* < 0.001 for hsCRP ≥ 3 mg/L) [24] and 53 OC-users athletes (OR = 13.3, 95% CI 4.14–42.6, *p* < 0.001 for hsCRP ≥ 3 mg/L) [25] and with a large Danish study finding low-grade inflammation (hsCRP 3–10 mg/L) in 29.9% of OC-users compared to 7.9% in non-OC users [27].

The role of hsCRP attesting low-grade inflammation in women was highlighted by large studies (41, 58). An American study demonstrated that women who developed cardiovascular events had higher baseline hsCRP levels than control subjects, so that hsCRP was a strong independent risk factor for any vascular event (RR = 4.8; 95%CI = 2.3–10.1) and for myocardial infarction or stroke (RR = 7.3; 95%CI = 2.7–19.9) [63]. Further studies confirmed the key role of chronic low-grade hsCRP for risk of future CVDs in women [41].

Recent evidence supports a role of chronic inflammation for female cancers [14,64,65]. Notably, combined estrogen plus progestogen contraceptives are considered human carcinogens and classified in Group 1 by the International Agency for Research on Cancer [14] for the liver and bile duct, breast and uterine cervix cancer.

Moreover, combined oral contraceptives may affect the mediators of low-grade chronic inflammation with potential additive risk in women with polycystic ovary syndrome (PCOS); however clinical implications of OC use by PCOS patients need further studies [30].

New evidence suggests that inflammation [16] and oxidative stress [36] are implicated in the aetiology of depression and disturbed sleep [66]; in turn, OC use has been associated with depression [17]. More longitudinal research is needed to improve the understanding of mechanisms induced by inflammation in OC-users possibly affecting the psyconeurological pathways [67].

### 4.3. Correlation between Oxidative Stress and hsCRP

Overall, our data highlighted a strong positive correlation of oxidative stress with hsCRP (*p* < 0.001) in healthy young women; women with oxidative stress over 400 FORT Units were about eight time as likely to have hsCRP ≥ 2.0 mg/L. So far, there has been a limited focus on this relationship specifically in women without pathologies.

There is scientific evidence demonstrating elevation of oxidative stress and inflammation in several pathological conditions, particularly of vascular nature [68]. Correlation between hsCRP and blood markers of oxidative stress was observed for instance in acute myocardial infarction [69] and in patients with high risk of CVD [70]. The novelty of our study is finding such a correlation in healthy subjects, suggesting that some general physiological mechanism links oxidative stress and low-grade inflammation even in the absence of evident pathologies. Such effects are likely mediated by factors like nuclear factor kappa-light-chain-enhancer of activated B cells (NF-kB) and nuclear erytroid 2 like factor-2 (Nrf2), two main transcription factors modulating several genes implicated both in oxidative stress and inflammation, through a complex balance among a variety of factors [71].

In our study causality cannot be inferred, because the design of the study does not allow to determine whether oxidative stress induces inflammation and/or whether the increase of hsCRP provokes hydroperoxidation. It is to mention, however, that the nuclear factors NF-kB and Nrf2 are key molecular switches both in oxidative stress and inflammation pathways [38,71], thus, molecular mechanisms could activate at the same time oxidative stress and inflammation.

In OC-users we found a higher frequency of elevated oxidative stress and inflammation compared to non-OC-users. dos Santos and colleagues [31] hypothesized that several mechanisms, including oxidative stress, elevate inflammation in hormonal contraception users, suggesting a causative role of oxidative stress in blood hsCRP increase. The biochemical pathways linking oxidative stress and inflammation elevation have, however, to be still completely elucidated, in particular in view of their potential adverse effects, including for example thromboembolic events, endothelial damage, CVDs, and cancer [50,72].

### 4.4. Impact of the Composition of the Contraceptive Pill

Accumulating evidence suggests that the progestin component of combined contraceptive pills may be important in determining the side effects of OC use [2,5,20]. Specifically, recent studies demonstrated that hormonal contraception can modify the redox status in the vasculature of women using combined contraceptive pills containing low doses of ethinyl-estradiol and progestin agents such as drospirenone [21,73] or norethisterone [19].

New generations of OC pills are characterized by lower estrogen content and by newer progestins, like desogestrel, gestodene, cyproterone, and drospirenone with lower androgenicity than past generation pills [59]. They have been introduced to reduce severe adverse effects of OC use, especially thromboembolism, and other cardiovascular diseases [11]. However, these new OC preparations are still associated with the risk of pulmonary embolism, myocardial infarction, thrombotic stroke and VTE [2,59]. A meta-analysis of observational studies [6] found that all four generations of progestin were associated with an elevated risk of ischemic stroke, and with a higher first-ever ischemic stroke risk associated with current OC use compared with non-current OC use (overall summary OR = 2.47, 95%CI = 2.04–2.99). The risk of ischemic stroke among current OC-users decreased significantly with decreasing estrogen dose: OCs of ≥50 μg ethinyl-estradiol (EE) had OR = 3.28 (95%CI = 2.49–4.32), 30–40 μg EE OR = 1.75 (95%CI = 1.61–1.89), 20 μg EE OR = 1.56 (95%CI = 1.36–1.79), whereas progestin only pills had not significant OR = 0.99 (95%CI = 0.71–1.37) [6].

A Danish study [2] examined 1,626,158 nonpregnant women, 15–49 years-old, with no history of CVD or cancer. As compared with non-users, current use of OCs was associated with an absolute increased risk of thrombotic stroke and myocardial infarction, ranging from 0.9 to 1.7 for OC containing 20 μg EE, and ranging from 1.3 to 2.3 for preparations with 30 to 40 μg EE, with relatively small differences in risk according to progestin type [2].

The risk of VTE associated to OC use is of particular concern and has been recently investigated in a total of 10,562 cases of thromboembolism [5]. In respect to no exposure to OCs in the previous year, exposure to OC containing desogestrel had increased risk with OR of 4.28, cyproterone 4.27, drospirenone 4.12, gestodene 3.64, norethisterone 2.56, norgestimate 2.53, and levonorgestrel 2.38 [5]. Similarly, another study [74] found that the relative risk of VTE for combined oral contraceptives with 30–35 μg ethinyl-estradiol and gestodene, desogestrel, cyproterone acetate, or drospirenone were similar and about 50%–80% higher than for combined oral contraceptives with levonorgestrel.

In our study we did not find statically significant differences in hsCRP values between users of second, third and fourth generation pills. However, second generation pill containing levonorgestrel had lower FORT values than third and fourth generation pills. Notably the group of OC containing desogestrel, cyproterone, and drospirenone had higher hydroperoxides values compared to all other progestins. A relevant issue for future investigations is to assess whether increased risk of thromboembolism in OC-users is mediated by the increased oxidative stress and/or chronic low-grade inflammation.

### 4.5. Strengths and Limitations

Limitations of our study include the recruitment of young adult Caucasian females, and thus, results cannot be generalized to older women and/or to women with different ethnic backgrounds; only women taking monophasic combined contraceptive pills were included in the study, excluding other types of contraceptive drugs; OCs were heterogeneous in type and amount of hormonal components although the majorities were OCs of third generation; detailed data about composition and dosing of potentially antioxidant supplements like vitamin E, C, and β-carotene were not registered [33,35].

Strengths of the present study include assessment of oxidative stress and hsCRP and several lifestyles and alimentary habits, the homogeneous ethnic group, the rather narrow age range of women, and strictly healthy subject inclusion. Of note, all study women were recruited before September 2019, thus avoiding any possible confounding by unrecognized SARS-Cov2 infection, although in our opinion it will be interesting in the future to explore the role of hormonal contraception in SARS-Cov2 infection.

## 5. Conclusions

This study adds to the existing evidence that OC use alters the oxidative homeostasis and modifies low-grade inflammatory status of young women. We found very high oxidative stress in the vast majority of OC-users (77% of them had FORT ≥ 400 Units). The strong positive relationship, that we observed in healthy women, between oxidative stress and basal chronic inflammation has several potential implications. Elevation of both parameters can potentially concur in OC side effects like the increased risk of CVDs, cancer and other diseases, posing the question of medical eligibility criteria for contraceptive use [75,76], in particular for women affected by major diseases like high risk of CVDs, having had cancer or with major immune/inflammatory diseases like multiple sclerosis [77], diabetes [45,78] or HIV infection [79,80]. It remains to be determined whether oxidative stress or hsCRP elevation is the main driver of CVD risk and whether these two conditions are synergistic.

We also investigated lifestyle and alimentary habits of study women. OC-users appear resilient to antioxidant food and low BMI protective effects. However, in our opinion, future studies on food supplements having antioxidant and anti-inflammatory roles [54,56,81,82] could be designed and especially targeted for OC-user women. Based on our data, concurrent use of pro-oxidant [82] and/or proinflammatory [83] drugs in addition to OC use should also be carefully taken into account, and will require future investigations. We think advisable that, at least in women with pathologic conditions and/or at high risk of developing a pathology, oxidative stress and hsCRP should be monitored over OC time use.

## Figures and Tables

**Table 1:** Comparison between the main characteristics of the OC-users vs. non-OC-users.

Characteristic	OC-Users ^a^ (*n* = 100)	Non-OC-Users ^a^ (*n* = 190)	*p* Value
Age ^a^ (years)	22.7 ± 3.50	23.4 ± 5.23	0.887 ^b^
Weight ^a^ (kg)	60.1 ± 7.59	60.3 ± 8.43	0.921 ^b^
Height ^a^ (m)	1.68 ± 5.94	1.69 ± 0.07	0.752 ^b^
Body-mass index ^a^ (kg/m^2^)	21.2 ± 2.24	21.2 ± 2.35	0.993 ^b^
University education, n (%)	89 (89.0)	151 (79.5)	**0.041** ^c^
Married, n (%)	5 (5.0)	19 (10.0)	0.142 ^c^
Nulliparity, n (%)	99 (99.0)	185 (97.4)	0.668 ^d^
Smokers, n (%)	21 (21.0)	27 (14.2)	0.139 ^c^
Cigarettes/day ^a^	0.83 ± 2.16	0.75 ± 2.68	0.166 ^b^
Coffee drinkers, n (%)	78 (78.0)	154 (81.1)	0.537 ^c^
Coffee cups ^e^/day	1.2 ± 1.02	1.5 ± 1.19	0.055 ^b^
Nutritional supplement use, n (%)	23 (23.0)	29 (15.3)	0.103 ^c^
Oxidative stress (FORT Units)	472 (403–532)	270 (229–308)	**<0.001** ^b^
hsCRP (mg/L)	1.31 (0.79–3.34)	0.35 (0.17–0.72)	**<0.001** ^b^

^a^ Values are mean ± SD or median (25th–75th percentile) as appropriate; ^b^ comparison of OC-users and non-OC-users by two-tailed Mann Whitney test; ^c^ comparison of OC-users and non-OC-users by two-tailed chi-square Pearson’s test; ^d^ comparison of OC-users and non-OC-users by two-tailed Fisher’s exact test as appropriate; ^e^ Italian espresso coffee cups. Statistically significant differences are highlighted by bold characters.

**Table 2:** Comparison of hydroperoxides and hsCRP values among the three different generation pills.

Biomarker	2° Generation OC-Users ^a^ (*n* = 10)	3° Generation OC-Users ^a^ (*n* = 55)	4° Generation OC-Users ^a^ (*n* = 35)	*p* Value ^b^ 2° vs. 3° Generation	*p* Value ^b^ 2° vs. 4° Generation	*p* Value ^b^ 3° vs. 4° Generation
Oxidative stress (FORT Units)	366 (332–434)	468 (416–530)	491 (434–566)	**0.009**	**0.008**	0.236
hsCRP (mg/L)	1.06 (0.83–3.05)	1.21 (0.70–3.39)	1.67 (0.86–3.34)	0.592	0.445	0.898

Second generation pill containing levonorgestrel; third generation pill containing gestodene and desogestrel; and fourth generation pills containing drospirenone, cyproterone, dienogest, clormadinone and nomegestrolo acetate. ^a^ Values are median (25^th^–75^th^ percentile); ^b^ comparison between generation pills by two-tailed Mann Whitney test. Statistically significant differences are highlighted by bold characters.

**Table 3:** Relative risk (OR) of OC-users vs. non-OC-users for categorical values of oxidative stress and hsCRP.

Measure	OC-Users (*n* = 100) N (%)	Non-OC-Users (*n* = 190) N (%)	OR ^a^ (95% CI)	*p* Value ^a^	Adjusted OR ^b^ (95%CI)	*p* Value ^b^
FORT ≥ 310 Units	92 (92.0)	45 (23.7)	37.1 (16.7–82.1)	**<0.001**	100 (30.1–334)	**<0.001**
FORT ≥ 400 Units	77 (77.0)	3 (1.6)	209 (60.9–715)	**<0.001**	342 (69.4–1688)	**<0.001**
hsCRP < 0.5 mg/L	16 (16.0)	118 (62.1)	0.12 (0.06–0.21)	**<0.001**	0.07 (0.03–0.16)	**<0.001**
hsCRP ≥ 2.0 mg/L	41 (41.0)	18 (9.5)	6.64 (3.54–12.4)	**<0.001**	11.1 (4.90–25.2)	**<0.001**
hsCRP ≥ 3.0 mg/L	33 (33.0)	13 (6.8)	6.71 (3.33–13.5)	**<0.001**	8.05 (3.47–18.7)	**<0.001**

FORT, oxidative stress expressed in FORT Units; hsCRP, high-sensitivity C-reactive protein; OR, odds ratio; CI, confidence interval. *p* value calculated by two-tailed chi-square Pearson’s or Fisher’s exact test as appropriate. ^a^ Univariable OR; ^b^ Adjusted for age, body-mass index, smoking, supplement use, and chocolate, fresh vegetable, fruits, red meat and fish servings per week; Statistically significant differences are highlighted by bold characters.

**Table 4:** Relative risk (OR) between two thresholds of elevated and low hydroperoxides for categorical hsCRP values among 290 women.

Measure	FORT ≥ 310 *n* = 137 n (%)	FORT < 310 *n* = 153 n (%)	OR (95%CI) *p* Value	FORT ≥ 400 *n* = 80 n (%)	FORT < 400 *n* = 210 n (%)	OR (95%CI) *p* Value
hsCRP < 0.5 mg/L	26 (19.0)	108 (70.6)	0.10 (0.06–0.17)**<0.001**	7 (8.8)	127 (60.5)	0.06 (0.03–0.14)**<0.001**
hsCRP ≥ 2.0 mg/L	49 (35.8)	10 (6.5)	7.96 (3.84–16.5)**<0.001**	38 (47.5)	21 (10.0)	8.14 (4.34–15.3)**<0.001**
hsCRP ≥ 3.0 mg/L	39 (28.5)	7 (4.6)	8.30 (3.57–19.3)**<0.001**	32 (40.0)	14 (6.7)	9.33 (4.62–18.8)**<0.001**

Two thresholds of elevated hydroperoxides levels (≥310 or ≥400 FORT Units) were compared with the corresponding low hydroperoxides levels (<310 or <400 FORT Units, respectively) in the whole group of 290 women. OR, odds ratio; CI, confidence interval; *p* value by two-tailed chi-squared test. Statistically significant differences are highlighted by bold characters.

**Table 5:** Correlations (r_s_) between continuous concentrations of oxidative stress and other lifestyle/alimentary biomarker continuous values.

Characteristic	All Women (*n* = 290) r_s_, *p* Value	OC-Users (*n* = 100) r_s_, *p* Value	Non-OC-Users (*n* = 190) r_s_, *p* Value
hsCRP (mg/L)	**0.622, <0.001**	**0.442, <0.001**	**0.426, <0.001**
Age, years	0.041, 0.489	0.047, 0.644	0.080, 0.274
Weight, kg	0.092, 0.122	−0.011, 0.910	**0.207, 0.005**
Height, cm	−0.013, 0.822	−0.103, 0.307	0.027, 0.715
BMI, kg/m^2^	**0.118, 0.047**	0.061, 0.547	**0.252, 0.001**
Cigarettes/day	0.084, 0.152	−0.002, 0.985	0.029, 0.696
Coffee cups ^a^/day	−0.099, 0.098	−0.118, 0.251	0.029, 0.701
Tea cups/day	0.079, 0.188	0.078, 0.447	−0.046, 0.540
Milk cups/week	0.042, 0.517 ^b^	0.135, 0.204 ^d^	0.032, 0.700 ^f^
Yogurt, 125 g servings/week	−0.035, 0.593 ^b^	−0.097, 0.364 ^d^	−0.053, 0.521 ^f^
Cheese, 50 g servings/week	−0.034, 0.602 ^b^	−0.006, 0.955 ^d^	0.040, 0.630 ^f^
Rice, 80 g servings/week	−0.031, 0.634 ^b^	0.130, 0.222 ^d^	−0.138, 0.094 ^f^
Pasta, 100 g servings/week	0.047, 0.473 ^b^	−0.122, 0.252 ^d^	0.103, 0.210 ^f^
Fruits, 200 g servings/week	**−** **0.136, 0.035 ^b^**	−0.167, 0.115 ^d^	−0.129, 0.116 ^f^
Tomatoe/eggplant/pepper, plates/week	0.009, 0.891 ^b^	−0.133, 0.224 ^d^	−0.008, 0.930 ^f^
Fresh vegetables, plates/week	−0.087, 0.180 ^b^	−0.066, 0.537 ^d^	−0.062, 0.453 ^f^
Legumes, plates/week	−0.023, 0.726	−0.111, 0.298 ^d^	0.029, 0.727 ^f^
Eggs, number/week	−0.002, 0.972 ^b^	−0.051, 0.641 ^d^	0.051, 0.542 ^f^
Red meat, 150 g servings/week	**−** **0.190, 0.004 ^b^**	−0.031, 0.777 ^d^	**−** **0.222, 0.007 ^f^**
Total meat (any type), 150 g servings/week	**−** **0.141, 0.029 ^b^**	0.022, 0.839 ^d^	−0.090, 0.273 ^f^
Sausages, 50 g servings/week	0.000, 0.995 ^b^	0.043, 0.686 ^d^	0.054, 0.511 ^f^
Fish, 200 g servings/week	**−** **0.143, 0.027 ^b^**	−0.039, 0.717 ^d^	**−** **0.164, 0.045 ^f^**
Sweet cakes, 50 g servings/week	−0.055, 0.393 ^b^	−0.025, 0.813 ^d^	0.072, 0.382 ^f^
Chocolate, 50 g servings/week	**−** **0.132, 0.041 ^b^**	−0.050, 0.640 ^d^	**−** **0.180, 0.028 ^f^**
Wine, 125 mL glasses/week	0.033, 0.596 ^c^	0.046, 0.665 ^e^	−0.005, 0.945 ^g^
Beer, 200 mL glasses/week	0.080, 0.199 ^c^	0.096, 0.360 ^e^	0.057, 0.466 ^g^
Spirits, 40 mL glasses/week	−0.008, 0.900 ^c^	−0.069, 0.510 ^e^	0.103, 0.190 ^g^

Two-sided Spearman correlations (r_s_) between continuous values of FORT Units and other biomarker continuous values are summarized for all women sample (*n* = 290), and separately for the 100 OC-users and the 190 non-OC-users. ^a^ Italian espresso coffee cups; ^b^ data available for 240 women; ^c^ data available for 258 women; ^d^ data available for 90 OC-user women; ^e^ data available for 93 OC-user women; ^f^ data available for 150 OC-user women; ^g^ data available for 165 non-OC-user women. Statistically significant differences are highlighted by bold characters.

## Data Availability

The datasets used and/or analysed during the current study are available from the corresponding author on reasonable request.

## Sample Availability

Not available.

## Abbreviations

BMI	Body mass index
CI	Confidence interval
CVD	Cardiovascular disease
FORT	Free oxygen radical test
hsCRP	High-sensitivity C-reactive protein
IQR	Interquartile range: from 25th to 75th percentile
NF-kB	Nuclear factor kappa-light-chain-enhancer of activated B cells
Nrf2	Nuclear erytroid 2-like factor-2
OC	Oral combined contraceptive
OR	Odds ratio
PCOS	Polycystic ovary syndrome
ROS	Reactive oxygen species
r_s_	Spearman’s Rho correlation coefficient
VTE	Venous thromboembolism
